# Analysis of the spatial distribution of cases of Zika virus infection and congenital Zika virus syndrome in a state in the southeastern region of Brazil: Sociodemographic factors and implications for public health

**DOI:** 10.1002/ijgo.13049

**Published:** 2020-01-23

**Authors:** Helaine J.S. Mocelin, Rafael C. Catão, Paula S.S. Freitas, Thiago N. Prado, Adelmo I. Bertolde, Marcia C. Castro, Ethel L.N. Maciel

**Affiliations:** ^1^ Laboratory of Epidemiology Federal University of Espírito Santo Vitória ES Brazil; ^2^ Graduate Program in Collective Health Federal University of Espírito Santo Vitória ES Brazil; ^3^ Departament of Geography Federal University of Espírito Santo Vitória ES Brazil; ^4^ Department of Statistics Federal University of Espírito Santo Vitória ES Brazil; ^5^ Department of Global Health and Population Harvard T.H. Chan School of Public Health Boston MA USA

**Keywords:** Brazil, Congenital Zika virus syndrome, Public health, Spatial distribution, Zika virus infection

## Abstract

**Objective:**

To perform spatial distribution analysis of reported cases of Zika virus and congenital Zika syndrome (CZS) in the state of Espírito Santo, Brazil, by neighborhood, and relate the results to sociodemographic indicators and implications for the health process.

**Methods:**

An ecological study using data from the 2016 National Notifiable Diseases Surveillance System, epidemiological records, and information on neighborhoods of families confirmed with CZS from qualitative field research.

**Results:**

Sociodemographic indicators were analyzed in three distinct groups: general population with Zika virus, pregnant women with Zika virus, and cases of CZS visited. For the three groups, average literacy rates were 71.1%, 71.0%, and 68.3%; the average income per minimum wage was 1.4, 1.1, and 1.4; sanitary sewage coverage was 75.6%, 76.1%, and 71.4%; garbage coverage was 90.8%, 91.2%, and 89.2%; and water supply was 93.8%, 94.1% and 93.8%, respectively. Socioeconomic indicators showed no significant differences between groups, although they were above the national average. A nonsignificant variation of 68.3%–71.1% was seen in the average literacy level above 15 years of age.

**Conclusion:**

Socioeconomic and demographic indicators of cases of Zika virus infection and CZS may indicate that the outbreak had different impacts according to class, social group, or gender, reflecting the persistence and social geography of inequality in Brazil.

## Abstract 2

The sociodemographic profile of cases of Zika virus and congenital Zika virus syndrome reveals different impacts on social strata and reflects the social geography of inequality in Brazil.

## 1 INTRODUCTION

Zika virus is a mosquito‐borne flavivirus transmitted mainly by *Aedes aegypti*; it was first identified in monkeys on the African continent in 1947 through a network that monitored yellow fever.^1^, ^2^ The first outbreak described in the literature occurred in 2007 in the Federated States of Micronesia, Oceania.^3^ In 2013, the virus began to spread to other parts of the continent.^4^, ^5^, ^6^, ^7^, ^8^ In mid‐2014 and 2015, Brazil became the epicenter of an epidemic, with confirmed exposure in all regions of the country.^9^

Since then, Zika virus infections have been reported in epidemic proportions, affecting more than 80 countries and subsequently causing a pandemic.^10^, ^11^ Countries most affected were those that had never had circulation of Zika virus (and thus the population was susceptible to infections), were endemic for dengue (*Ae. aegypti* was found), and contained areas with conditions often associated with arbovirus transmission (e.g. high population density, ideal climatological conditions, lack of infrastructure). It has also been speculated that extreme climatic conditions may have been a contributor to Zika virus spread.^12^

Zika virus symptoms (similar to dengue) include fever, skin rash, conjunctivitis, muscle and joint pain, and malaise or headache, with a duration of 2–7 days. In Brazil, Zika virus infection was marked by a finding never before described in the scientific literature: fetal microcephaly.^13^ The perception of the increase in the number of microcephaly cases in newborns in northeastern Brazil in 2015 prompted the need for studies and research to elucidate the history of the disease and the behavior of the virus. From 2000 to 2014 the average annual number of cases of microcephaly in Brazil was 164, whereas in 2015 alone there were 1608 cases.^14^, ^15^ Such an increase could not be explained by genetic factors; exposures to other diseases such as syphilis, toxoplasmosis, rubella, *Cytomegalovirus* and herpes simplex infections (STORCHZ); severe malnutrition; or exposure to harmful substances (alcohol, certain drugs, or toxic substances).^16^ Microcephaly was found to be just one of the manifestations of an anomaly called congenital Zika syndrome (CZS).^17^, ^18^ Conditions associated with CZS include eye damage, joint problems, excessive muscle tone, and seizures, among other signs and symptoms.^1^, ^19^

In view of the harmful consequence of microcephaly, in November 2015 the Ministry of Health declared a Public Health Emergency of National Concern; in February 2016, the World Health Organization (WHO) declared a Public Health Emergency of International Concern.^20^, ^21^ These emergencies ended in May 2017 and November 2016, respectively.^22^

In February 2016, Zika virus infection became a disease of mandatory reporting, leading to increased investment in research on the disease.^1^, ^23^, ^24^ Following improvements in epidemiological surveillance, research on the association between Zika virus infection and dissemination patterns of the disease has become more robust.^25^ In addition, areas were affected in an unequal way, with a higher incidence of CZS infection in places of social vulnerability.

Accordingly, important government measures were implemented to address issues surrounding poverty and inequity to access, namely the supply of insect repellent to pregnant women in the public service and monthly payment of the Continuous Cash Benefit, corresponding to approximately 1‐month earnings at minimum wage (US$ 247.96) for children diagnosed with CZS.^26^, ^27^

According to the Ministry of Health, between epidemiological weeks 45 of 2015 and 52 of 2018, 17 041 suspected cases of changes in growth and development, possibly related to Zika virus infection and other infectious etiologies, were reported. The incidence of Zika virus infection reported in the state of Espírito Santo was high, with 440 cases reported during the same period.^28^

The United Nations Food and Agriculture Organization (FAO) maps critical zones and groups more prone to virus infection, and maps zones as they are likely to control infection. FAO can also make use of models with meteorological, socioeconomic, environmental information and data, and provide an efficient and intersectoral response to fight infection.^29^, ^30^

Mapping of diseases helps to describe their spatial behavior, creating clues for the development of public practices and policies focused on health care and surveillance. The maps also allow correlation of various factors that are present in the territory such as infrastructure, living conditions, and inequalities. Analysis of the spatial pattern of Zika virus makes it possible to identify the most affected areas and the main social determinants of the disease.^31^

Information gaps for the country, and the state of Espírito Santo in particular, remain for the Zika virus epidemic and socio‐environmental factors. The aim of the present study was to describe the spatial distribution of Zika virus and CZS cases in the state of Espírito Santo according to neighborhoods, and to describe the sociodemographic indicators and their implications in the health–disease process.

## 2 MATERIALS AND METHODS

We conducted an ecological study with a descriptive approach using secondary data from the National Notifiable Diseases Surveillance System (SINAN) for the year 2016 when the highest incidence of cases of Zika virus infection were reported in Brazil. The analysis of CZS cases in Espírito Santo from January 1, 2015 to December 31, 2016 was based on data from the epidemiological records of the Health Department of the state of Espírito Santo (SESA‐ES), as well as information provided by the neighborhoods of families to a field research project with a qualitative approach, of which the present study formed part. During the field research, data were collected at the homes of mothers of children with CZS, with the purpose of describing their sociodemographic profile, as well as analyzing their perception of the diagnosis, the conditions of care, and their child's daily care. A data collection form was used to identify the sociodemographic profile. As a complementary method of data collection, a field diary was also used to record the observations inherent to the place and housing conditions during the visits to the mothers’ homes.^32^

Regarding mapping of cases on a national scale, Zika virus notification data in the general population and in pregnant women were obtained, spatially aggregated per unit of the federation, from the SINAN database. For analysis at a regional scale, Zika virus cases were mapped in the general population using the rate per 100 000 inhabitants, and in the case of pregnant women, absolute numbers were used due to the absence of a denominator for the calculation of rates; the figures were spatially aggregated per neighborhood. The neighborhood of residence field was used as a spatial aggregation unit for the reported cases of Zika virus infection. This unit was available on the basis of the most disaggregated and was an intermediary between the municipality and the place of residence.

ArcGIS (ESRI, Redlands, CA, USA) software was used for geo‐referenced data, with the federal unities’ cartographical bases, municipalities, and census tracts available from the Brazilian Institute of Geography and Statistics (IBGE). The neighborhoods were obtained from the Jones of Santos Neves Institute, a state‐level research institute. The reported cases were added to the base in a georeferenced environment, distinguishing the cases of pregnant women from the others. This spatial unit aggregation allows a higher level of scale refinement than municipality, indicating possible spatial clusters within municipalities. Sociodemographic variables such as general water supply network, garbage collection service, sanitary coverage through general sewage network, total monthly income, and literacy over 15 years of age were obtained from the 2010 census. We used the geometric growth method to correct the population (from 2010 to 2016), applying the verified growth of the municipalities in the neighborhoods.^33^

For this aggregation unit, there were no datasets available with the same territorial delimitation, except for census tract data (grouping of 300 households on average), which did not hold exact geographic correspondence with the neighborhoods. There were cases where some census tracts crossed two or more neighborhoods, and in other places there was a total absence of census tracts in some areas of the neighborhoods. To adjust this situation and minimize data loss we added in a geographical information system (GIS) layer of spatial information containing sociodemographic variables in a raster format. This kind of file format is a data matrix in which data are stored in each pixel with a determined size (smaller than a census tract), allowing topological and spatial operations with fewer errors. In this operation we divided a census tract area into smaller pieces (20 × 20 meters), which allowed us to recompose the socioeconomic data in the neighborhoods, merging the smaller pieces of data using means and standard deviations. For the total population, however, the sectors were proportionally added in the neighborhoods by means of operation between layers with zonal statistics also using the raster. The overall incidence rate was calculated with the estimated population by neighborhoods.

CZS cases in Espírito Santo were georeferenced by municipalities due to the incompleteness of the information in the neighborhood field (about 93%). However, it was possible to map CZS cases by neighborhood that were visited during the field research cited above. The sociodemographic information of the neighborhoods was collected for these interviewees, using the census variables.

The research project was submitted to the Research Ethics Committee of the Health Sciences Center of the Federal University of Espírito Santo (CEP/CCS/UFES) and approved with Opinion 1 730 231 from 09/09/2016. It was also approved by the International Ethics Committee (PAHOERC) under PAHO‐2017‐02‐0013.

## 3 RESULTS

In Brazil, 268 805 Zika virus cases were reported in SINAN. Of those, 181 142 (67.4%) were women, 24 143 (9.0%) were pregnant women, and 10 769 (4.0%) had Zika virus infection confirmed by positive blood or urine examination by polymerase chain reaction (PCR) or serology. Among the total number of reports, 342 did not indicate gender.

**Figure 1:**
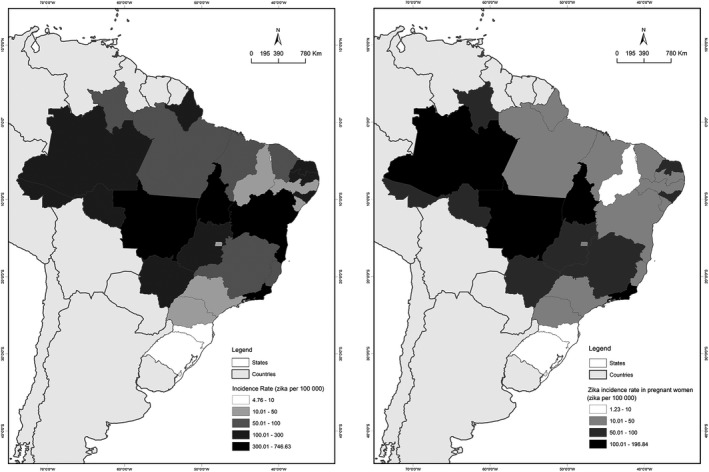
Spatial distribution of the incidence of Zika virus in the general population and pregnant women in Brazil from the National Notifiable Diseases Surveillance System (SINAN), 2016.

The Brazilian states that had the highest incidence of Zika cases in 2016 were Rio de Janeiro 75 977 (28.3%), Bahia 57 066 (21.2%), Mato Grosso 24 680 (9.2%), and Tocantins 6209 (2.3%), representing 61.0% of all Brazilian cases. Moreover, 14 908 (2.4%) cases were observed in rural areas of the country. The state of Espírito Santo reported 2780 (1.0%) Zika virus cases; 7109 (2.6%) reports had this variable missing (Fig. 1).

The spatial distribution of pregnant women was mainly concentrated in the states of Rio de Janeiro (n=5722, 23.7%), Mato Grosso (n=1778, 7.4%), Amazonas (n=1293, 5.4%), and Tocantins (n=428, 1.8%) (Fig. 1). The majority of women with Zika virus infection declared to be non‐white (n=72 071, 65.0%) or white (n=38 787 (35.0%). A total of 70 284 (38.0%) cases had left this variable blank. Women of childbearing age (15–49 years) accounted for 124 783 (68.9%) of all females.

Regarding education, only 63 664 (35.1%) women had this information in SINAN. Of these, 873 (1.4%) were illiterate; 5757 (9.0%) had incomplete first to fourth grade; 3481 (5.5%) had completed fourth grade (former primary education); 9462 (14.9%) had incomplete fifth to eighth grade; 5024 (7.9%) had completed elementary education; 8003 (12.6%) had incomplete high school; 20 540 (32.3%) had completed high school; 3274 (5.1%) had incomplete higher education; and 7250 (11.0%) had completed higher education (Table 1).

**Table 1:** Education level of female Zika virus cases in Brazil reported in the National Notifiable Diseases Surveillance System (SINAN) in 2016

Education	No. (%)
Illiterate	873 (1.4)
First to fourth grades incomplete	5757 (9.0)
Up to fourth grade complete	3481 (5.5)
Fifth to eighth grade incomplete	9462 (14.9)
Up to eighth grade complete	5024 (7.9)
Incomplete high school	8003 (12.6)
Complete high school	20 540 (32.3)
Incomplete higher education	3274 (5.1)
Complete higher education	7250 (11.0)

In the state of Espírito Santo, 2774 cases of Zika virus infection were reported in 2016, of which 1939 (69.9%) were women and 324 (16.7%) were pregnant women. Of these pregnant women, 193 (59.6%) cases were confirmed by positive blood or urine test for Zika virus infection by PCR or serology, according to SINAN data, and the remaining were only clinically diagnosed. The infection occurred in 58 municipalities in the state.

**Figure 2:**
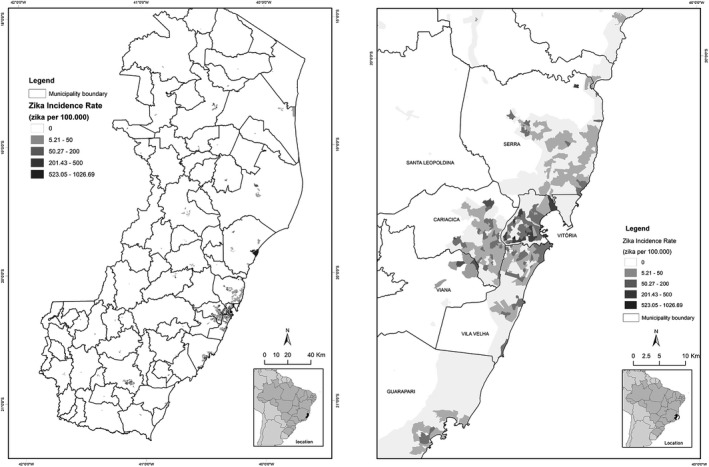
Spatial distribution of notifications of Zika virus infection in the general population according to municipality of residence, in the state of Espírito Santo, data from the National Notifiable Diseases Surveillance System (SINAN), 2016.

The municipalities with the highest incidence of reports were Vitória with 1069 (38.5%) cases, Vila Velha with 402 (14.5%), Cariacica with 358 (12.9%), Serra with 272 (9.8%), and Cachoeiro de Itapemirim with 226 (8.1%) cases (Fig. 2). These municipalities correspond to the five most populous regions of the state, which together represent 48.5% of the total population of the state.

Cases of women of childbearing age (15–49 years) in the state totaled 1374, which accounted for 70.9% of all reports among females. The majority declared to be non‐white (n=645, 57.3%), while 486 (43.2%) women declared to be white; in 814 reports this variable was not reported. Regarding education, 873 (45.0%) women had this information in the SINAN form. Of these, 5 (0.6%) were illiterate; 56 (6.4%) had incomplete first to fourth grade; 31 (3.6%) had completed fourth grade (former primary education); 128 (14.7%) had incomplete fifth to eighth grade; 140 (16.0%) had completed elementary education; 113 (12.9%) had incomplete high school; 364 (41.7%) had completed high school; 63 (7.2%) had incomplete higher education; and 172 (19.7%) had completed higher education. For 123 (6.4%) it did not apply and 750 (38.7%) were left blank (Table 2).

**Table 2:** Education level of female Zika virus cases in the state of Espírito Santo in the National Notifiable Diseases Surveillance System (SINAN) in 2016

Education	No. (%)
Illiterate	5 (0.6)
First to fourth grades incomplete	56 (6.4)
Up to fourth grade complete	31 (3.6)
Fifth to eighth grade incomplete	128 (14.7)
Up to eighth grade complete	140 (16.0)
Incomplete high school	113 (12.9)
Complete high school	364 (41.7)
Incomplete higher education	63 (7.2)
Complete higher education	172 (19.7)
Not applicable, blank, or ignored	873 (45.1)

**Figure 3:**
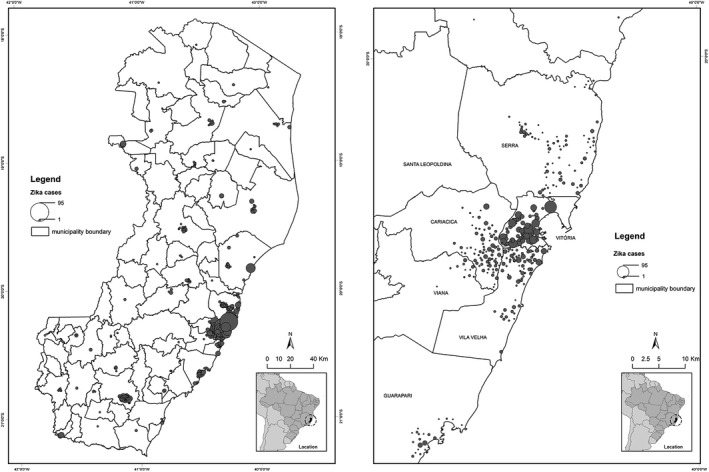
Spatial distribution of notifications of pregnant women with Zika virus in the state of Espírito Santo from the National Notifiable Diseases Surveillance System (SINAN), 2016.

The spatial distribution of pregnant women reported with Zika virus in the state of Espírito Santo showed that 28 municipalities reported the disease. Most cases occurred in the metropolitan region of Vitória, which includes Cariacica (n=64, 19.8%), Fundão (n=3, 0.9%), Guarapari (n=14, 4.3%), Serra (n=43, 13.3%), Viana (n=7, 2.2%), Vila Velha (n=52, 16.0%), and Vitória (n=60, 18.5%), totaling 243 cases and representing 75.0% of all cases of pregnant women reported. Eight (2.5%) cases were reported in rural areas of the state (Fig. 3).

With regard to CZS in the state of Espírito Santo, 49 cases were confirmed according to data from the epidemiological records of SESA‐ES. The municipalities with the highest incidence were Cariacica with 11 (22.4%) cases; Aracruz with 7 (14.3%) cases; Serra, Vitória, and Vila Velha with 5 (10.2%) cases; Guarapari with 4 (8.2%) cases; Cachoeiro de Itapemirim with 3 (6.1%) cases; Linhares, São José do Calçado, and Viana with 2 (4.0%) cases; Alto Rio Novo with 1 (2.0%), Mountain with 1 (2.0%), and Muqui with 1 (2.0%) case.

**Figure 4:**
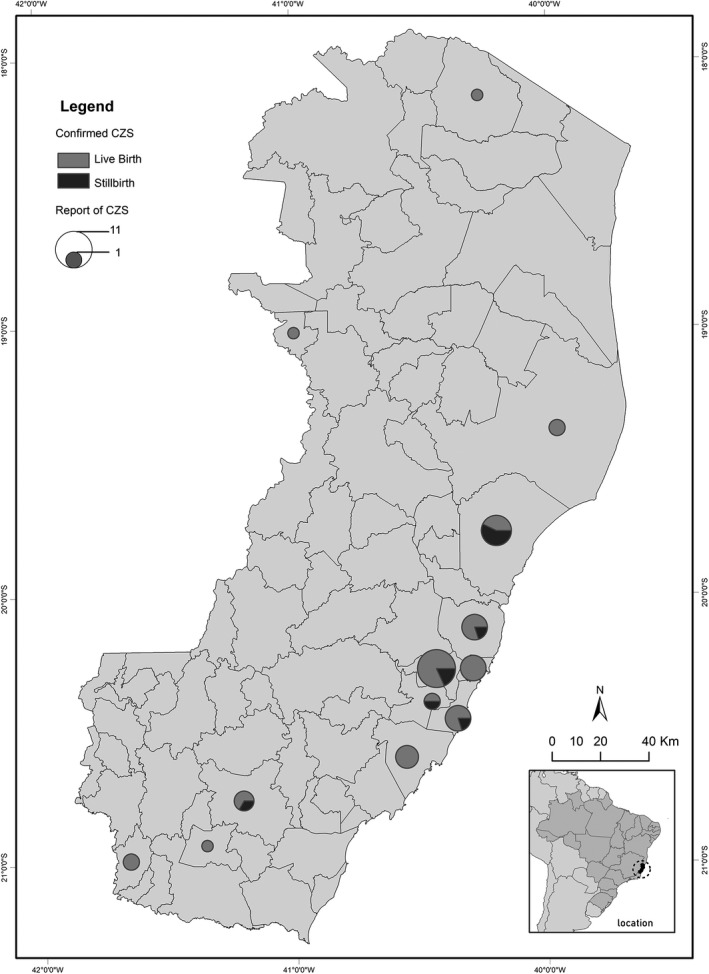
Spatial distribution of reported cases and number of deaths from congenital Zika syndrome in the state of Espírito Santo from 2015–2016 according to Health Department of the state of Espírito Santo (SESA‐ES) epidemiological records.

Of these 49 confirmed cases of CZS, 10 died (Fig. 4). A total of four deaths occurred in the city of Aracruz, two in Cariacica, and one case in each of the municipalities of Cachoeiro de Itapemirim, Serra, Viana, and Vila Velha.

From those cases, it was possible to map 25 per neighborhood visited during the field research. For these interviewees, the sociodemographic information of the neighborhoods was collected using the census variables. The socioenvironmental variables addressed were general water supply network, garbage collection service, and sanitary coverage through general sewage network. The information concerning social aspects included nominal monthly income per household and education over 15 years.

Average socioeconomic and demographic indicators were analyzed in three groups, namely, total number of reports of Zika virus infection, pregnant women with Zika virus infection, and families visited with confirmed cases of CZS; the average literacy rate over 15 years was 71.1%, 71.0%, and 68.3%; the average total nominal monthly income per household in minimum wages was 1.4, 1.1, and 1.4; average sanitary coverage through the general sewage network was 75.6%, 76.1%, and 71.4%; coverage by garbage collection service was 90.8%, 91.2%, and 89.2%; and the supply by general water network was 93.8%, 94.1%, and 93.1%, respectively (Table 3).

**Table 3:** Averages for socioeconomic indicators in the state of Espírito Santo from the 2010 census with extracts of reports Zika virus infection cases in the year 2016 per neighborhood

Groups	Literacy, %	Income^a^	Sewage, %	Garbage, %	Water, %
Total reports of Zika virus infection	71.1	1.4	75.6	90.8	93.8
Pregnant women reported with Zika virus infection	71.0	1.1	76.1	91.2	94.1
Congenital Zika syndrome visited cases	68.3	1.4	71.4	89.2	93.1

a Income refers to the average total nominal monthly income per household in minimum wages for 2010. At that time, the minimum wage was about 510.00 Brazilian Reais or approximately US$ 126.00.

## 4 DISCUSSION

Results from the present study show that the Zika virus epidemic primarily affected women of childbearing age both nationally and in the state of Espírito Santo. Pregnant women affected by the Zika virus were predominantly non‐white and had completed high school. Most Zika virus/CZS notifications occurred in cities, with few notifications in rural areas. Averages for socioeconomic indicators in Espírito Santo from the 2010 census, by neighborhood and according to three groups (general population with Zika virus, pregnant women with Zika virus, and CZS cases visited), showed similarity between the groups, with indicators considered acceptable.

Several studies are in line with our results showing that the Zika virus epidemic in Brazil mostly impacted women.^34^, ^35^ The high prevalence of affected women in the SINAN‐NET databases can be explained in part by similar findings from a serological survey conducted in Micronesia.^1^, ^3^ The rate of attack of clinical cases of fever caused by Zika virus in women in Micronesia was high; however, the prevalence of positive IgM serology was higher in males, with a relative risk of 1.1 in relation to females.^1^, ^3^ This may suggest that asymptomatic infections are more frequent in males.^36^

The results of the present study also demonstrate the predominance of non‐white race/color in the reports of Zika virus infection in Brazil, where it is strongly linked to poverty and a lower social status compared with the white population.^37^ Those with low socioeconomic status usually live in places with poor infrastructure that favors the reproduction of the Zika virus vector. In this sense, the present findings have strengthened the association between poverty and higher incidence of the disease.

Data on gender, percentage of pregnant women reported, education, and race/color found in Brazil are similar to data from the state of Espírito Santo. Despite the low completeness of the variables for eduction and race/color, Zika virus cases were more frequent in women who had completed high school and were brown‐skinned.

Regarding the socioeconomic indicators, it is important to highlight that poverty and inadequate infrastructure such as lack of access to treated water and poor sanitation are factors that may contribute to a higher risk of disease transmitted by vectors to vulnerable segments of the population. The base of evidence linking social determinants of health, such as poverty and social or geographical marginalization, to infectious diseases (e.g. malaria, tuberculosis, and Ebola) continues to grow.^38^ Like other mosquito‐borne diseases such as dengue and Chikungunya, Zika is not distributed randomly or equally in the total population.^39^

It is important to highlight that the control of *Ae. aegypti* is one of the main challenges, considering its fundamental role in the transmission of Zika virus. In Brazil, especially in large cities, the elimination of *Ae. aegypti* breeding sites is a complex task, particularly where precarious housing conditions, intermittent water supply, inadequate sanitation, and uneven garbage collection exist.^40^

Averages for socioeconomic indicators in Espírito Santo from the 2010 census, by neighborhood, showed no significant differences between the three groups for sewage, garbage coverage, and water, but were all above the national average.^31^ However, other studies point to Zika virus and CZS with social determination rooted in poverty and environmental and housing conditions.^34^, ^41^, ^42^ A qualitative study by Freitas et al.,^32^ with 25 mothers of children with CZS in the state, reinforces these inequalities by describing the residence scenarios as peripheral areas with precarious conditions, such as houses built on unprotected slopes and with imminent risk of collapse, wooden houses, house with unpainted and unfinished masonry, floors consisting of bare earth, neighborhoods without access to garbage collection and without sewage connection.

These contradictory findings can be explained by the socioeconomic indicators in the state, which has the fifth highest rate in Brazil—at 76.8%—of population living in houses with simultaneous access to the three basic sanitation services (direct or indirect garbage collection, water supply by general network, and sewage by collection or pluvial network).^31^ However, average monthly household income does not seem to reflect income inequality in the country and state. The average total monthly income per household per capita in the state of Espírito Santo, by neighborhood, did not vary significantly between the three groups. The average income of the three groups ranged from 1.1 to 1.4 minimum wages per household for 2010; it is noteworthy that Brazil does not have an official definition to characterize poverty; however, to receive government social assistance, household income per person needs to be below one quarter (0.25) of current minimum wage.^33^

Income inequality is high in Brazil. According to the National Household Sample Survey (PNAD), the average per capita income of 20% of the households with the highest incomes (R$ 4499.15 or approximately US $1117.80) was 18.3 times higher than the average income of the 20% with lowest incomes (R$ 243.60 or approximately US $60.37) in 2016.^31^

In Espírito Santo, the average monthly income of all workers was R$ 1962 (US $487.47), approximately 1.9 minimum wages in the year 2016. However, more than half of the state's population earn less than one minimum wage and the vast majority receive even less than this.^31^

A study carried out in the state with 25 families affected by CZS showed that the household income per capita was up to R$ 500 for 80% of respondents.^32^ Studies in other states also showed average household income per capita of R$ 266.00 (US $61.61) and R$ 400.00 (US $99.38).^43^

Regarding the average literacy level above 15 years in the three groups, our study showed a variation of 68.3%–71.1%, with no significant difference between the groups. These values differ from the average rate in Brazil of approximately 93.0%, and of 93.8% in the state of Espirito Santo.^44^

Regarding the implications of the study, based on the assumption that approximately 10% of all Zika virus notifications were among pregnant women, implementing preventive measures for pregnant women, improving women's access to health services, and improving maternal health care—including family planning, prenatal diagnosis services, and the dissemination of accurate and complete information—are all crucial.^45^

Although the mosquito is the main link in the transmission of Zika virus, other modes of transmission need to be considered, particularly sexual transmission, which is the second most important form of virus spread. Therefore, involving men in preventing the spread of Zika virus and preventing pregnancy in areas at risk of Zika infection should be considered.^30^ Health professionals should expand information to both men and women, thus avoiding gender inequality in responsibility for combating the epidemic.

A limitation of the study is that the results do not consider the social heterogeneity of the neighborhoods given the use of averages for comparison. Due to the lack of spatial adequacy between layers (census tract and neighborhoods) we had to adapt the data per neighborhood that were homogenized using averages, especially for population. In addition, the transformation of census tract information into neighborhoods can have an effect known as the “modifiable areal unit problem” (MAUP) whereas the neighborhood boundaries can distort the census data. Another important limitation concerns the demographic data for race/color and education, which had a high proportion of nonresponses.

The present study did not correlate the SINAN‐NET database with the database of the Public Health Events Registry (RESP). Therefore, we cannot determine the percentage of pregnant women registered in SINAN‐NET who had babies with congenital malformations or describe the profile of these women. Our analyses were restricted to the profile of symptomatic pregnant women with Zika virus that were registered in SINAN‐NET.

In conclusion, the profile of Zika virus infection cases reported in Brazil and in the state of Espírito Santo suggests that this epidemic may have a strong relationship with social and health inequalities present in the country. Socioeconomic and demographic indicators of Zika virus infection and CZS in the state may indicate that the outbreak had different impacts according to class, social group, or gender, reflecting the persistence and social geography of inequality in Brazil.

These data suggest that the underprivileged may be more susceptible to exposure to the virus, which can help to trigger action to promote public policies that support the reduction of social and gender inequalities, such as investment in education for vulnerable populations, as well as promotion of health education, employment opportunities, and income for women. In addition, healthy cities should be focused on urban planning and environmental sanitation, particularly regarding access to clean water, garbage collection, and sanitation. Large intersectoral investments aimed at improving the living conditions of the Brazilian population, especially in urban areas, are needed to combat *Ae. aegypti*—the vector of the disease.

In view of the magnitude of the Zika virus outbreak and the complications associated with CZS, further studies and more robust health information systems that identify more detailed census units are necessary to allow for the monitoring and spatial distribution of CZS cases and their sociodemographic indicators.

## AUTHOR CONTRIBUTIONS

HJSM, ELNM, and PSSF designed and conducted the survey. HJSM, ELNM, PSSF, RCC, TNP, AIB, FP, and MCC conducted a data review and wrote the article. All authors analyzed and interpreted the data, critically reviewed the content, and approved the final version.

## CONFLICTS OF INTEREST

The authors have no conflicts of interest.
